# Thoracolumbar Retrolaminar Block: Anatomical and Radiological Study of Injectate Pattern Distribution in Canine Cadavers

**DOI:** 10.3390/ani13193008

**Published:** 2023-09-25

**Authors:** Julia Pentsou, Michail Vagias, Thomas Davies, Séamus Hoey, Vilhelmiina Huuskonen

**Affiliations:** 1 Department of Veterinary Anaesthesia and Analgesia, Royal (Dick) School of Veterinary Studies, University of Edinburgh, Edinburgh EH25 9RG, UK; 2 Department of Small Animal Surgery, Royal (Dick) School of Veterinary Studies, University of Edinburgh, Edinburgh EH25 9RG, UK; mvagias2@ed.ac.uk; 3 Bristol Vet Specialists, Unit 10, More Plus Central Park, Madison Way, Severn Beach, Bristol BS35 4ER, UK; daniel.davies@bristolvetspecialists.co.uk; 4 Equine Clinical Studies, Diagnostic Imaging and Anaesthesia, UCD School of Veterinary Medicine, University College Dublin, D04 W6F6 Dublin, Ireland; seamus.hoey@ucd.ie (S.H.); vilhelmiina.huuskonen@ucd.ie (V.H.)

**Keywords:** analgesia, dogs, spinal surgery, hemilaminectomy, retrolaminar block

## Abstract

**Simple Summary:**

The analgesic management of dogs with spinal disease is important, as insufficient pain relief may result in chronic neuropathic pain. The retrolaminar block is a relatively new local anaesthetic technique, first developed in humans, in which the local anaesthetic is deposited into the musculofascial plane on the lamina of the target thoracic vertebra, thus providing analgesia to the thoracic region. The aim of this study was to evaluate the retrolaminar technique as a peri-operative analgesic option for dogs with spinal disease, by determining the distribution of two volumes of contrast-dye mixture and the potential complications after anatomical landmark-guided thoracolumbar retrolaminar injections in canine cadavers. Eight canine (greyhound) cadavers were randomised into two groups that received either a 10 or a 20 mL injection into the retrolaminar space at the level of the twelfth thoracic vertebra, after which they underwent computed tomography and anatomical dissection to determine the spread of the injectate. The injectate was detected in the surrounding intervertebral foramina and the paravertebral and retrolaminar fascial planes, which could suggest promising analgesic potential for spinal pain. Epidural and retroperitoneal spread was also noted, and the spread was not volume-dependent. Further studies will help to determine the exact indications for this technique.

**Abstract:**

The retrolaminar block is a regional anaesthetic technique, first developed in humans, in which the local anaesthetic is deposited directly onto the dorsal aspect of the thoracic or lumbar vertebral lamina. This study aims to evaluate the distribution of landmark-guided thoracolumbar retrolaminar injections in greyhound cadavers. Thirteen injections of contrast-dye solution were performed in eight cadavers at the level of the twelfth thoracic vertebra (T12), with either 20 mL (n = 8, high volume, HV) or 10 mL (n = 5, low volume, LV) per site. The spread of the injectate was evaluated through computed tomography and transverse anatomical dissection. The groups were compared using the Mann–Whitney U test. The median (range) of the extent of the spread was 4 (2–5) and 3 (2–4) intervertebral foramina in the LV and HV groups, respectively. The median (range) of the spread along the retrolaminar space was 3 (2–3) retrolaminar segments in the LV and 3 (2–4) in the HV group. Epidural and retroperitoneal spread was identified in seven cadavers. Following landmark-guided retrolaminar injections, the injectate spread both in the retrolaminar and paravertebral spaces, without any obvious association between the volume of injectate and the extent of the spread. Further studies are warranted to determine the clinical efficacy of the technique.

## 1. Introduction

Multimodal analgesia regimens commonly include, where possible, a regional anaesthetic technique that is, ideally, minimally invasive, technically easy, and has a very-low-to-zero complication rate [1]. Furthermore, the worsening opioid crisis [2], which will potentially impact global opioid availability, has led researchers worldwide to focus on finding alternatives in analgesic management for both humans [3] and veterinary patients [4].

**Figure 1:**
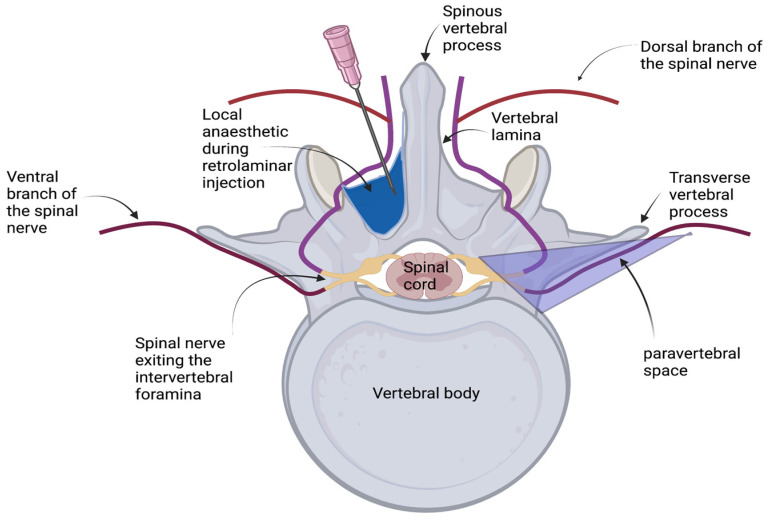
Depiction of the retrolaminar space in comparison with the paravertebral space. During the retrolaminar injection, the local anaesthetic is deposited directly on the target vertebral lamina, into the retrolaminar plane, which is the plane between the dorsal aspect of the vertebral lamina and the overlying paraspinous muscles (blue area), without the needle ever entering the thoracic paravertebral space (purple area) (created by JP with BioRender.com).

Fascial plane blocks are novel regional anaesthetic techniques that rely on the spread of local anaesthetic within the fascial plane, where a peripheral branch of the target nerve runs [5]. A fascial plane block called the retrolaminar block was described for the first time in 2006 as an easier and safer alternative approach to the thoracic paravertebral block in humans [6]. During the retrolaminar block, the local anaesthetic is deposited directly on the target vertebral lamina, into the retrolaminar plane, which is the plane between the dorsal aspect of the vertebral lamina and the overlying paraspinous muscles [7], without the needle ever entering the thoracic paravertebral space (Figure 1). The thoracic paravertebral space (TPVS) was described in humans as “a triangular-shaped space located bilaterally alongside the whole length of the thoracic vertebral column” [8] and is delineated by the internal intercostal membrane on the dorsal aspect, the parietal pleura on the ventrolateral aspect, and the vertebral body, the intervertebral disc, and the intervertebral foramen at the medial aspect [9]. The analgesic effect of the retrolaminar block is not yet fully understood [10], but is believed to be a combination of the passive spread of the local anaesthetic to the thoracic paravertebral and epidural spaces, and the blockade of the dorsal branch of the spinal nerves that cross the retrolaminar plane [1]. Therefore, the intensity of the clinical effect of the retrolaminar block depends on the passive spread of the local anaesthetic from the injection point, thus limiting the risk of pleural injury or direct nerve damage [10].

Since the first description of the retrolaminar block, several other researchers have explored the technique, with or without the aid of ultrasound, either via anatomical investigation [7,10,11,12] or in clinical studies [13,14,15,16,17,18,19]. In humans, the retrolaminar block has steadily gained popularity and has been used in the analgesic management of rib fractures [14,18], thoracic surgeries [20], mastectomies [13], inguinal herniotomies [21], and abdominal laparoscopic surgeries [17,22], all with promising results. Recently, a bilateral retrolaminar block in a man was used successfully to provide analgesia for lumbar vertebral surgery [23]. 

The performance of the landmark-guided retrolaminar block begins with the identification of the spinous process of the vertebra of interest, followed by the insertion of the needle through the surrounding paraspinal muscles, in a parasagittal plane, until it comes into contact with the dorsal aspect of the vertebral lamina, which is where the local anaesthetic is injected [13]. Recently, a case report described the successful application of the thoracolumbar retrolaminar block in seven dogs that underwent spinal surgery [24]. To the authors’ knowledge, this is the first study that investigates the spread of the injectate following a retrolaminar injection in canine cadavers. 

The objectives of this study were to evaluate the distribution pattern of two different injectate volumes following a thoracolumbar retrolaminar injection, with the help of computed tomography and transverse anatomical dissection. The hypothesis was that the spread of the injectate to and within the paravertebral space, and within the retrolaminar space, would depend on the volume injected, with higher volume spreading to a greater extent along both spaces. 

## 2. Materials and Methods 

This study was granted an exemption from full ethical review by the Animal Research Ethics Committee of University College Dublin (AREC-E-20-08-Huuskonen). 

### 2.1. Animals and Study Design

Eight frozen adult greyhound cadavers, five males and three females, euthanised for reasons unrelated to this study, were used after being thawed at ambient temperature for 72–96 h. The number of cadavers was chosen according to previous, similar studies. Any cadavers with spinal abnormalities that could have affected the identification of the anatomical landmarks were excluded from the study. Three cadavers were randomly allocated to receive a unilateral right or left retrolaminar injection to evaluate the potential spread into the contralateral paravertebral space (two right and one left injection), while the remaining five cadavers received bilateral injections. The thirteen injections were further randomised into either 10 mL (low-volume injection group, LV, n = 5) or 20 mL (high-volume injection group, HV, n = 8) of a 1:20 mixture of methylene blue (Methylene blue, 1%, Merck, Germany) and iodinated contrast (ioversol) (Optiray^®^, 300 mg I/mL, Guerbet, France), respectively. Both randomisations were achieved via the removal of a ballot from an opaque envelope containing eight and thirteen ballots, respectively. All thirteen retrolaminar injections were performed at the level of the 12th thoracic spinal vertebra (T12). The distribution of the injectate was evaluated using computed tomography in all cadavers and transverse anatomical dissection in two cadavers. 

### 2.2. Retrolaminar Injections

All cadavers were weighed and placed in sternal recumbency for the injections. The wings of the ilium and the spinous process of the sixth lumbar vertebra were located via palpation, and the lumbar and thoracic spinous processes were palpated cranially and marked with a skin marker. Following this, two investigators, blinded to each other, confirmed the position of the 12th thoracic vertebra in each cadaver (JP, MV). 

**Figure 2:**
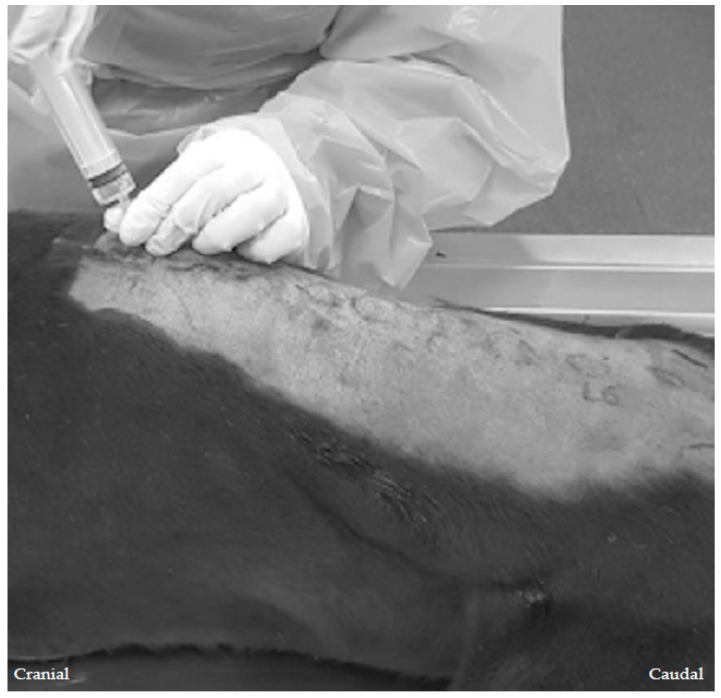
The landmark-guided approach to the twelfth thoracic (T12) retrolaminar space in a canine cadaver. With the cadaver in sternal recumbency, the spinous processes are palpated until the T12 spinous process is identified. An 18-gauge 1-inch needle is introduced next to the spinous process through the epaxial muscles, orientated caudoventrally, into a parasagittal plane, and towards the retrolaminar space—the space between the bony lamina and the muscles that surround it—until contact with the T12 vertebral lamina is achieved. The needle remains strictly in the parasagittal plane, parallel to the dorsal spinous process, and at a 45° angle to the skin, to minimise the risk of inadvertent intrapleural or epidural injection. Once the needle is in the target space, the injection of the contrast-dye mixture is performed.

An 18-gauge, 1-inch needle (Disposable injection needles, Kruuse, Denmark), connected to a 20 mL syringe (Omnifix, Braun Medical, Germany) containing the iodinated contrast and methylene blue dye mixture, was introduced into the epaxial muscles, in a parasagittal plane, approximately 1–1.5 cm lateral to the spinous process, at an angle of 45° to the skin. The needle was then orientated caudoventrally, and advanced until contact with the vertebral lamina was achieved. The advancement of the needle was then stopped and, following a negative aspiration for air and blood, the retrolaminar injections were performed by the same investigator (JP). The depth of the needle was approximately 1.5–2 cm, depending on the body condition score of the cadaver (Figure 2). 

### 2.3. Computed Tomography 

A computed tomographic (CT) examination of the thoracolumbar area was performed approximately twenty minutes after the injection of the contrast-dye mixture, using a 16-slice CT scanner (SOMOTO Scope: version syngo CT VC40, Siemens, Germany). The cadavers were positioned in sternal recumbency for the scan. The acquisition parameters used to obtain the scans were 0.75 mm slice thickness at 220 mAs and 130 KVp. The computed tomographic images were then viewed using image analysis software (OsiriX, Version 6.0; Pixmeo, Switzerland). The two EVCDI-board-certified veterinary radiologists who evaluated the CT images (SH, TD) were blinded to the technique used, but not to each other. 

The CT features assessed for each retrolaminar injection were the confirmation of injection at the T12 retrolaminar space (yes/no), the presence of contrast in the underlying ipsilateral paravertebral space (yes/no), the identification of the ipsilateral intervertebral foramina affected (cranial and caudal to T12), the extent of the contrast distribution in the ipsilateral retrolaminar space, the contrast distribution pattern (linear/intercostal), an epidural contrast spread (yes/no), the extent of the epidural contrast spread if any, the presence of contrast in the pleural space (yes/no), the presence of contrast in the retroperitoneal space (yes/no), the presence of contrast in the peritoneal space (yes/no), the presence of pneumothorax (yes/no), intravascular contrast (yes/no), and severe abnormalities of the vertebral column that could have hindered correct needle placement. The confirmation of injection at the T12 retrolaminar space was based on image analysis and the evaluation of the maximum volume of injectate at the point of injection. We considered a craniocaudal spread of the injectate along the paraspinous muscles as a linear contrast distribution pattern, and a more lateral spread towards the intercostal muscles as an intercostal distribution pattern.

### 2.4. Transverse Anatomical Dissection

Following the CT scan, two randomly selected cadavers were dissected using a band saw (BR360, MEDOC, Logroño, Spain). On both occasions, the parietal pleura and pleural cavity were carefully inspected for any evidence of needle penetration and contrast spread following the removal of the thoracic and abdominal cavity contents. Transverse plane sections of 2.5 cm width were performed on the first cadaver, while the width was adjusted to 1.5 cm on the second cadaver to enhance the detail. The sections were performed on the thoracolumbar area of the cadavers, from the level of the tenth thoracic vertebra (T10) to the level of the third lumbar vertebra (L3), with the target retrolaminar space identified. Photographs were taken, and the presence of methylene blue dye in the retrolaminar space of interest was recorded to assist in further evaluation.

### 2.5. Statistical Analysis

Statistical analysis was performed using IBM SPSS 27 (IBM Statistics; Albany, NY; USA) for macOS. The normality of the data was assessed using the Shapiro–Wilk test. Each hemithoracic retrolaminar injection was analysed as an independent variable. A comparison of the number of intervertebral foramina stained and retrolaminar spread with HV or LV was performed using a Mann–Whitney U test. All the statistical test results were interpreted using a 5% level of significance. The results are summarised as the median (range). Categorical data are presented as frequencies. 

## 3. Results 

### 3.1. Retrolaminar Injections

The median (range) weight of the canine cadavers was 29.55 kg (26.3–36.4 kg), with a median (range) body condition score of 4.5 (3.5–5) out of 9 [25]. The median (range) injection volume in the HV group was 0.67 mL/kg (0.55–0.76 mL/kg), and in the LV group was 0.36 mL/kg (0.3–0.38 mL/kg). All the injections were successfully performed in a single attempt. No further complications were noted during the procedure. 

### 3.2. Computed Tomography

**Figure 3:**
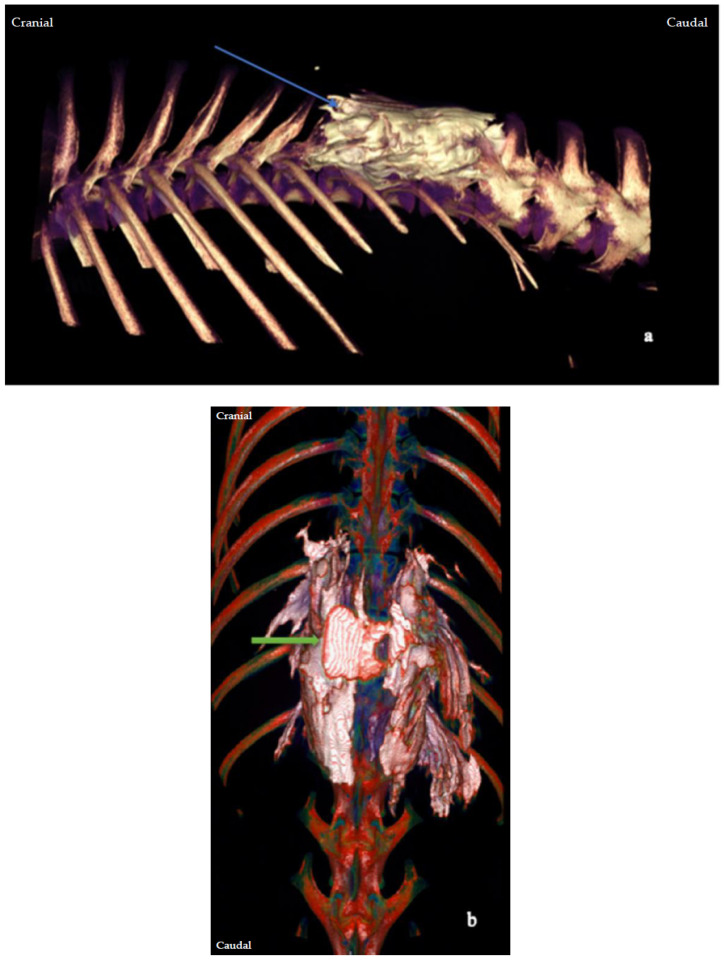
Cadaver 6. Three-dimensional volume-rendered computed tomography (CT) images of the thoracolumbar area of a canine cadaver, acquired after a retrolaminar injection at the level of the twelfth thoracic vertebra. (**a**) The lateral view. The blue arrow indicates the distribution of the contrast agent, which surrounds the retrolaminar space in the thoracolumbar area and correlates with the injection pathway. The image offers visual confirmation of the correct performance of the T12 retrolaminar injection, as well as an overview of the extent of the injectate spread. (**b**) The dorsoventral view of the same cadaver. The green arrow indicates the T12 point of injection and confirms the correct identification of the T12 retrolaminar space.

**Figure 4:**
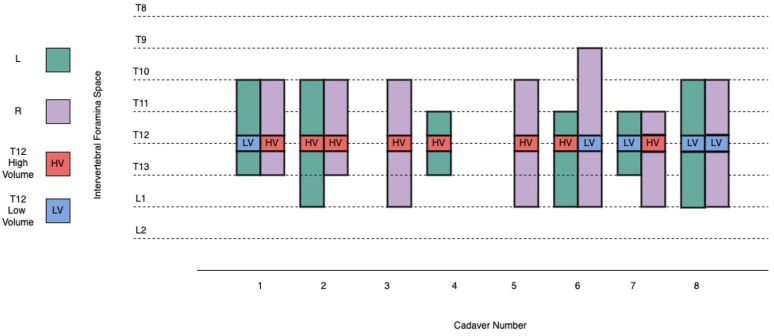
Description of the craniocaudal extent of the spread of the contrast-dye injectate into the thoracolumbar intervertebral foramina within the paravertebral space, in eight canine cadavers, following retrolaminar injections at the level of the twelfth thoracic vertebra (T12). The blue colour represents the point of the T12 retrolaminar injection in the low-volume group, and the red colour represents the point of the T12 retrolaminar injection in the high-volume group, as confirmed via CT scan. The green colour represents the left-sided spread of the injectate cranial and caudal to T12 (left-sided injection was performed in cadavers 1, 2, 4, 6, 7, and 8) and the purple colour represents the right-sided spread of the injectate cranial and caudal to T12 (right-sided injection was performed in cadavers 1, 2, 3, 5, 6, 7, and 8). CT = computed tomography, L = left, R = right, T12 = twelfth thoracic vertebra, LV = low volume, HV = high volume.

The CT study confirmed the presence of iodinated contrast injectate at the T12 retrolaminar space after all 13 injections. Contrast was identified in the ipsilateral paravertebral space on all (13/13) occasions, regardless of the volume used. The contrast distribution pattern was always both linear and intercostal (Figure 3). From the level of the T12 vertebral body, the median (range) cranial and caudal spread was 4 (2–5) intervertebral foramina for the low-volume group and 3 (2–4) intervertebral foramina for the high-volume group, with no statistically significant difference (*p* = 0.524) (Figure 4). 

In 7 out of 8 cadavers, epidural contrast spread of a varying extent was noted. While, on six occasions, it was limited locally to the injection site, in one cadaver (with 10 mL injectate volume) the epidural migration was quite extensive (T4–L4). The median (range) spread of the epidural injectate involved 3 (0–14) segmental levels. Focal presence of contrast in the retroperitoneal space was also noted in 12 out of 13 injections, but no specific path could be identified. There was no contrast present in the pleural space or the peritoneal space in any of the cadavers. Pneumothorax was identified in all eight cadavers. Intravascular contrast contamination was noted in 8 out of 13 injections, and it involved vessels away from the needle’s pathway, such as the azygos vein. No injectate was identified in the contralateral retrolaminar or paravertebral spaces in the three retrolaminar injections that were performed unilaterally. No abnormality of the vertebral column was identified in any of the cadavers (Table 1).

### 3.3. Transverse Anatomical Dissections

**Figure 5:**
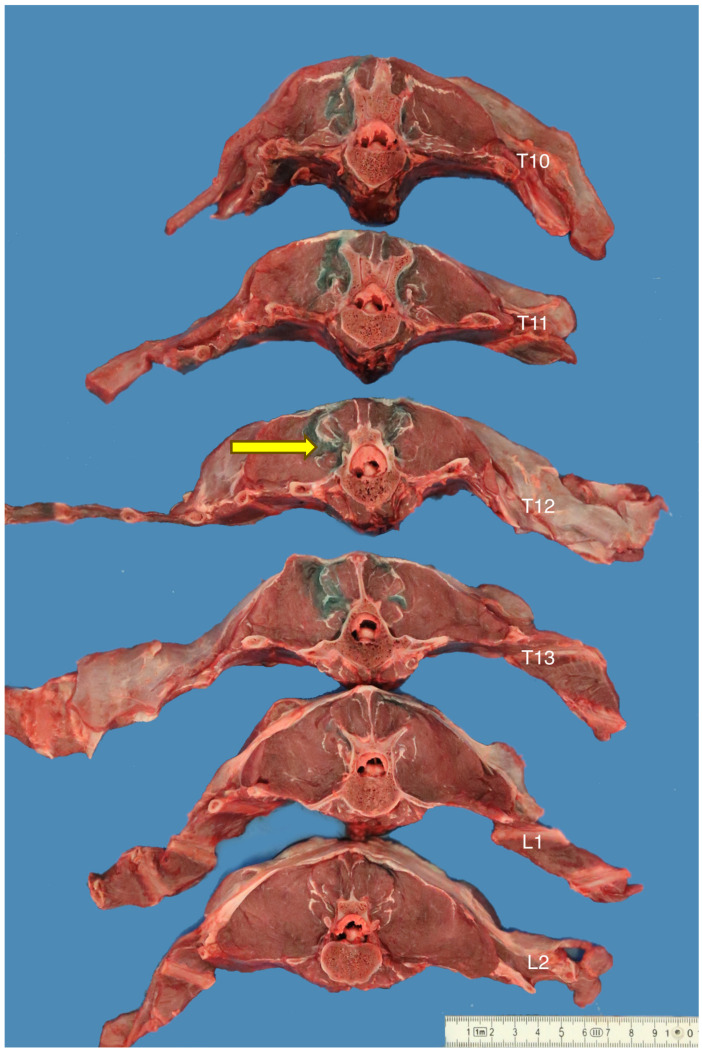
Transverse plane sections of the thoracolumbar spine of an adult greyhound cadaver (cadaver 1) following retrolaminar injections of contrast-dye solution at the level of the twelfth thoracic (T12) vertebra. The left side of the cadaver was injected with a low-volume injection (10 mL), while the right side was injected with a high-volume injection (20 mL). The yellow arrow depicts the dye surrounding the T12 intervertebral foramina on the left side.

Transverse anatomical dissection of two cadavers corroborated the CT study findings regarding correct T12 identification during the retrolaminar injection, the spread of the injectate into the retrolaminar spaces of interest, and the presence of dye in the underlying paravertebral space (Figure 5). No evidence of needle penetration or contrast contamination was noted in the pleural space of the two cadavers. Due to the highly diluted methylene blue dye, no further analysis was possible.

## 4. Discussion

This experimental cadaveric study describes a landmark-guided thoracolumbar retrolaminar technique in canine cadavers and evaluates the extent of the injectate spread into the paravertebral and retrolaminar spaces, while comparing two different volumes of injectate.

Contrary to our hypothesis, the spread of the injectate to and within the paravertebral space was not directly proportional to the volume used. Conversely, a previous study in pig cadavers reported that lower volumes of injectate did not spread to the paravertebral space, as assessed via anatomical dissection [11]. Our unexpected finding is perhaps due to the low number of injections performed, preventing us from detecting a proportional association between the volume used and the injectate spread. Ideally, a power calculation would have identified the number of cadavers required to examine such a relationship. However, due to the limited availability of canine cadavers for the study, we utilised eight cadavers, a number commonly used in similar studies [9,26]. Furthermore, the difference in our findings compared to the pig cadaver study could also be attributed to our use of advanced imaging (CT), which, as other authors believe [27], is a more sensitive tool to evaluate contrast spread, as the transverse slices produced via CT are substantially thinner that the ones produced via transverse anatomical dissection. Besides the obvious variability between different species, the pig cadavers were also fresh, whereas our canine cadavers were frozen and thawed and, therefore, our injectate spread could have potentially been affected by decreased tissue integrity.

The contrast spread noted in the ipsilateral retrolaminar space and the underlying ipsilateral paravertebral space supports both proposed theories about the analgesic mechanism of the retrolaminar block. The first theory suggests that the retrolaminar block is paravertebral by proxy. In previous human studies, it was hypothesised that the local anaesthetic diffused into the paravertebral space either through the porous costo-transverse ligament [6,13] or through the facet joints [14]. The second theory proposes that the distribution of the local anaesthetic into the fascial plane between the dorsal surface of the lamina and the epaxial muscles leads to the blockade of the dorsal cutaneous branch of the spinal nerve [1]. Based on the CT images showing the injectate surrounding the target intervertebral foramina, entering the paravertebral and epidural spaces, and spreading through the path of the dorsal branch of the spinal nerve in the retrolaminar space, a retrolaminar injection with local anaesthetic should provide analgesia through a combination of the aforementioned mechanisms. This distinct mode of action differentiates the retrolaminar block from the simple intramuscular infiltration of local anaesthetic into the thoracolumbar muscles. Epaxial infiltration has been investigated in dogs undergoing spinal surgery and has shown contradicting results: in one study, it was found to provide both surgical and postoperative analgesia [28], while in another study, intramuscular local anaesthetic did not enhance post-operative analgesia [29].

The vast majority of the retrolaminar techniques described in the human medical literature are performed at the level of the fourth thoracic vertebra. As over 50% of thoracolumbar disc lesions in dogs are found either between the T12 and T13 or the T13 and L1 [30] intervertebral spaces, the T12 space was chosen for our study. A retrolaminar block performed at the T12 level has the potential to provide peri-operative thoracolumbar analgesia in dogs undergoing hemilaminectomies. The volumes of injectate selected for our study were extrapolated from previous human [7] and porcine [11] cadaveric studies to ensure sufficient spread to the target nerves.

Epidural spread of a varied extent was also noted in seven cadavers. The previously published findings in humans are not consistent regarding the epidural migration of the injectate; some studies presenting it as a consistent occurrence and part of the exerted analgesic action of the retrolaminar technique [7], while others do not [11]. In those studies, the retrolaminar injections were performed at the level of the fourth thoracic vertebra, where the epidural spread of local anaesthetic could elicit severe complications, such as a sympathetic trunk blockade resulting in refractory hypotension [31], or further cranial epidural migration leading to potential respiratory failure [32] and arrhythmogenic effects [33]. In our study, the median epidural spread involved only three segmental levels, and extensive spread was seen only in one cadaver; however, in clinical patients even a single occurrence of extensive epidural spread could pose a major concern. While highly unlikely, the possibility of inadvertent epidural injection cannot be completely excluded in the one cadaver with an extended epidural spread. The incorporation of ultrasound guidance, smaller volumes of injectate, and the use of a blunter needle could potentially decrease the associated complication rates.

This study also revealed the presence of injectate in the retroperitoneal space of the cadavers. The retroperitoneal injectate was noted only focally, and no path leading to the retrolaminar space could be specified. Recently, a retrolaminar block with multiple injection points was performed to provide postoperative analgesia in humans who had undergone a retroperitoneal laparoscopic nephrectomy [34]. The block resulted in significantly lower pain scores, lower incidence of postoperative nausea and vomiting, earlier mobilisation, and reduction in plasma inflammatory factor levels. The authors speculated that the visceral analgesic effect was due to the spread of the local anaesthetic either in the paravertebral or in the epidural space. Future studies in dogs could potentially elucidate the implication of this finding and provide further details on whether the retrolaminar technique could be utilised to provide analgesia in retroperitoneal procedures.

Intervertebral disc disease is a prevalent spinal disorder in dogs that necessitates careful analgesic management [35], as it is associated with severe nociceptive, inflammatory, and neuropathic thoracolumbar pain [36]. The analgesic management of such complicated pain states involves the utilisation of several classes of drugs, each targeting a specific part of the pain pathway; this practice is commonly known as multimodal analgesia [37]. The drugs commonly incorporated in the treatment of thoracolumbar pain in dogs include full mu-agonist infusions, gabapentin, and pregabalin [38,39]. At the same time, regional anaesthetic techniques are considered an integral part of any carefully tailored analgesic plan and, as local anaesthetics are the only drugs that can achieve complete loss of pain sensation, their utilisation is strongly advised in every surgical case when possible [40]. One of the major benefits of regional anaesthetic techniques is their potential to decrease the requirement for other analgesics and anaesthetic drugs [41], and this may help to maintain intraoperative normotension [42]. The maintenance of normotension in dogs that suffer from thoracolumbar intervertebral disc disease and acute spinal cord injury is of the utmost importance, especially as hypotension is a commonly seen intraoperative complication in spinal surgeries [43], and several studies have demonstrated that the maintenance of normotension improves clinical outcome [44,45,46].

The thoracic paravertebral block (TPVB) is a regional anaesthetic technique that is well-described in veterinary literature [47,48] as an option to provide thoracic analgesia, although the technique requires thorough anatomical knowledge and training in order to be performed safely [11]. In humans, the TPVB has been associated with serious complications, such as pneumothorax and inadvertent epidural and intrathecal drug administration and, thus, the retrolaminar technique was developed as a safer alternative to the TPVB in an attempt to minimise complication rates [6,13].

Recently, a fascial plane block called erector spinal plane (ESP) block [49,50] has been successfully used for the analgesic management of thoracolumbar pain in dogs undergoing surgery for hemilaminectomy. This technique, however, necessitates the use of ultrasound to locate the target landmark, which is the tip of the transverse process of the ninth thoracic [51] or the fifth thoracic [26] spinal vertebra, which lies adjacent to the pleural space. Unfortunately, though, ultrasound is not a modality found in every veterinary practice [18] and, even when it is available, the performance of an ultrasound-guided regional anaesthetic technique still requires a level of technical training and thorough anatomical and sono-anatomical knowledge. Therefore, we believe that the landmark-guided thoracolumbar retrolaminar technique could have a place in the veterinarian’s arsenal. Likewise, in humans, fascial plane blocks have gained popularity in a very short period of time, partly because they are accessible by less experienced practitioners and, thus, provide the opportunity for more patients to benefit from locoregional analgesia [5].

An inherent limitation of this study is its cadaveric nature and the potential loss of tissue integrity that accompanies the post-mortem state, as decomposition has a detrimental effect on the physical properties of tissues [52]. This could have affected the diffusion pattern of the injectate and could potentially account for the epidural and intravascular migration of the contrast-dye mixture. Pneumothorax was also a consistent finding in all cadavers, with volumes of air so large (several litres) that it could not be justified by a single injection, and with no evidence of contrast in the pleural space. The large volumes of free air radiographically detected in the pleural cavity of canine cadavers are believed to be a result of tissue putrefaction [53]. Potentially, the use of fresh cadavers, as opposed to frozen and thawed cadavers, could have reduced some of these limitations. Furthermore, fresh cadavers would have allowed for a more accurate determination of negative aspiration for blood and air prior to injection, a step that could further enhance the safety of the technique, but such resources were not available to us.

All the cadavers used in this study had similar anatomical conformation, and none of them had a body condition score higher than 5 out of 9. A high body condition score could potentially obscure the anatomical landmark identification [54]. Furthermore, cadavers with anatomical spinal abnormalities were excluded from this study. Anatomical differences, such as spinal vertebral fractures, could limit the usefulness of the landmark-guided technique in dogs by preventing landmark identification. On top of that, all the cadavers utilised in this study were greyhounds; thus, we cannot determine whether the findings of this study could be extrapolated in other canine breeds with different anatomical characteristics.

We also noted injectate in the intravascular space, in vessels such as the azygos vein, in 8 out of 13 injections. Direct intravascular injection into the azygos vein during the performance of the retrolaminar technique is anatomically impossible, so this finding is either a consequence of the cadaveric loss of tissue integrity or, less likely, due to the high injection pressures as a result of the high viscosity of the contrast-dye mixture. However, we used large-bore hypodermic needles to deliver the contrast-dye mixture, which reduced the pressure during injection. Similar to our findings, a CT study of a paravertebral brachial plexus block in canine cadavers identified intravascular contamination in 33% of the subjects [27]. This has not been a common finding in previous cadaveric studies that investigate nerve blocks, as anatomic dissection is the usual method chosen to determine injectate distribution. However, CT imaging is potentially more sensitive in determining such details. Furthermore, in human patients undergoing radical mastectomy, arterial levobupivacaine plasma concentrations were below the toxicity threshold levels after landmark-guided retrolaminar catheter placement and continuous infusion of levobupivacaine post-operatively [15]. When clinically relevant local anaesthetic doses are used in vivo, and negative blood aspiration is performed prior to injection, the potential complications and intravascular spread should be limited.

Another limitation of the current study involves our use of iodinated contrast and methylene blue mixture, and the potential difference in the spread of this injectate compared to a local anaesthetic. The injectate distribution findings arising from any cadaveric study do not necessarily correlate with the clinical effects when the same technique is performed in live subjects [7], but cadaveric studies are commonly used as the first step when investigating a new regional anaesthetic technique [10].

Similar studies have mostly used anatomical dissection to assess the extent of nerve staining [55]. The extent of nerve staining is usually translated to the degree of analgesic efficacy of the technique. However, we believe that the CT study offered a more detailed examination of our hypothesis and helped us to evaluate the extent of injectate spread in the retrolaminar, paravertebral, and epidural spaces, as these anatomical regions are difficult to access with anatomical dissection without affecting the dye spread. Likewise, we chose not to re-freeze the cadavers prior to the transverse anatomical dissection, as was done in similar studies [11], as repeated cycles of thawing–freezing can affect muscle tissues and could also affect the spread of the injectate [52].

## 5. Conclusions

Following the performance of a novel landmark-guided thoracolumbar retrolaminar technique in canine cadavers at the level of T12, a proportional association between the volume injected and the spread of the injectate was not noted. The injectate was detected in the surrounding intervertebral foramina and the paravertebral and retrolaminar fascial planes, which suggests promising potential for spinal analgesia. The retrolaminar technique could perhaps be used in the future as part of a multimodal analgesic regime for canine thoracolumbar pain, provided that further studies confirm the safety and efficacy of this technique.

## Figures and Tables

**Table 1:** Description of the spread of the injectate as identified via the computed tomographic study to the surrounding areas, i.e., the retrolaminar space, the space surrounding the intervertebral foramina, and the epidural space.

Injection	Cadaver	Injection Volume (mL)	Retrolaminar Spread	Intervertebral Foramina Spread	Epidural Spread
1	1	20	T10–T13	T10–T13	T11–T13
2	1	10	T10–T13	T10–T13	T11–T13
3	2	20	T10–T13	T10–T13	L1
4	2	20	T10–T13	T10–L1	L1
5	3	20	T10–L1	T10–L1	T11–T13
6	4	20	T11–T13	T11–T13	None
7	5	20	T10–T13	T10–L1	T11–T13
8	6	10	T11–L1	T9–L1	T11–T12
9	6	20	T11–L1	T11–L1	T11–T12
10	7	20	T11–L1	T11–L1	T6–L1
11	7	10	T11–T13	T11–T13	T6–L1
12	8	10	T11–L1	T10–L1	T4–L4
13	8	10	T11–T13	T10–L1	T4–L4

## Data Availability

The data presented in this study are available in detail on request from the corresponding author.
